# Supplementary material for: Quantifying economic resilience from input–output susceptibility to improve predictions of economic growth and recovery
Source: Nat Commun. 2019 Apr 11;10:1677. doi: 10.1038/s41467-019-09357-w (PMC6459978; doi:10.1038/s41467-019-09357-w)
Supplement: Supplementary file 1 — Supplementary Information [file 41467_2019_9357_MOESM1_ESM.pdf]

Supplementary Information

for

Economic resilience from input–output susceptibility improves predictions of economic growth and recovery

Peter Klimek et al.

Supplementary Figure 1

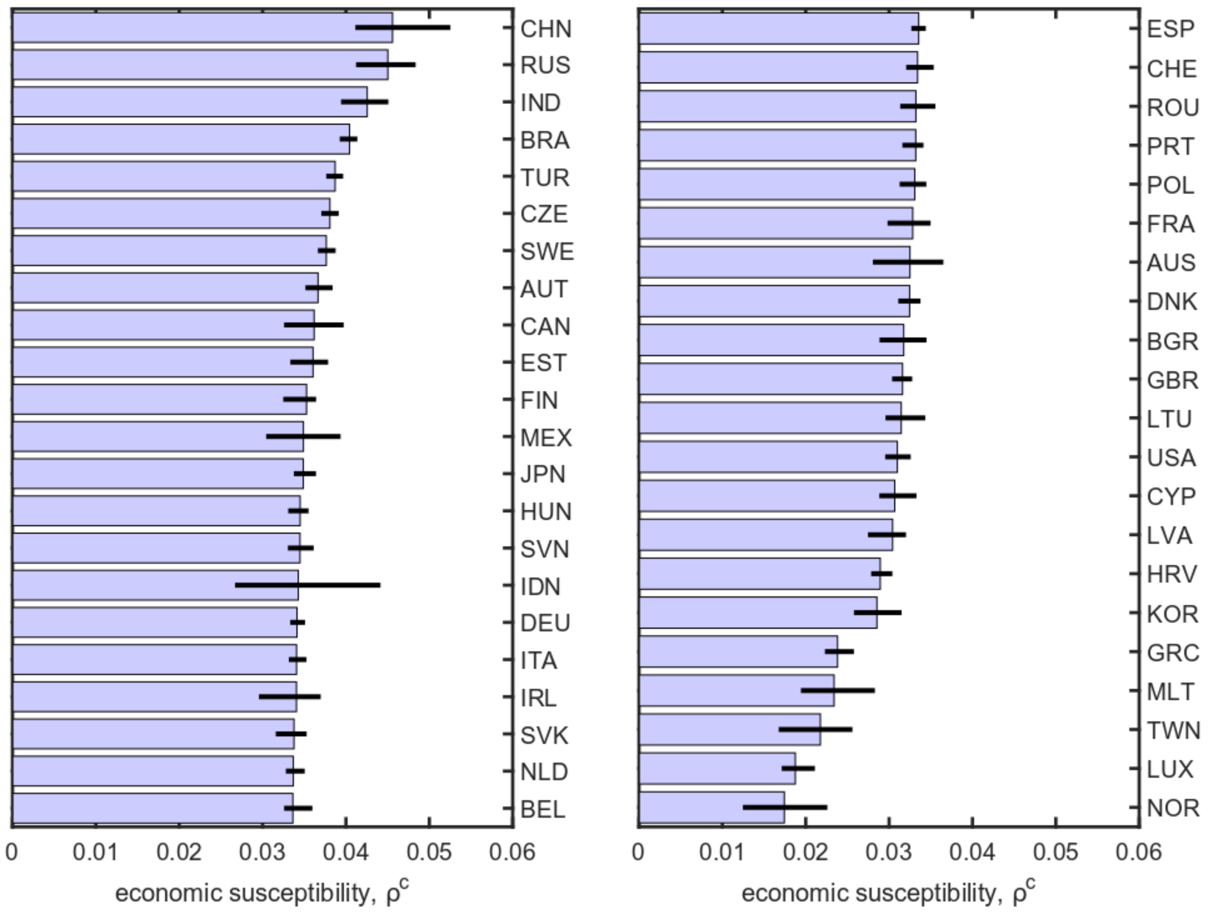

Ranking of countries according to their susceptibility to economic shocks. Country susceptibility is calculated as the average over the susceptibility of each of the country's sectors. Error bars indicate the standard deviations of the sectoral susceptibilities. Source data are provided as a Source Data file.

Supplementary Figure 2

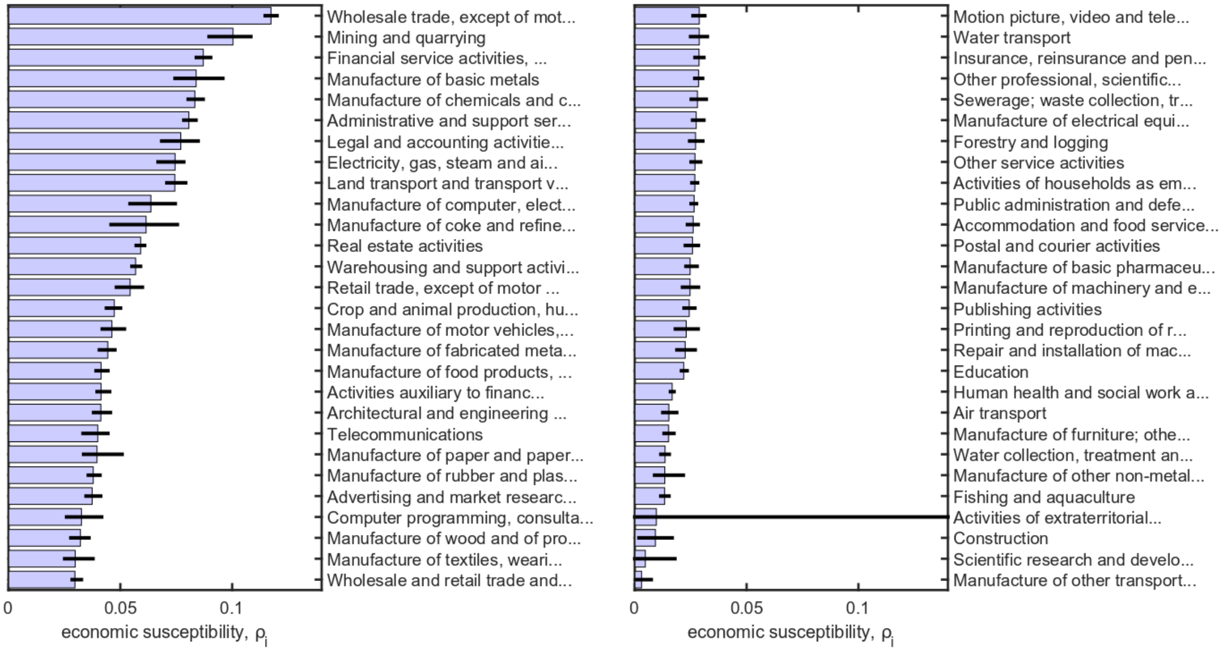

Ranking of sectors according to their susceptibility to economic shocks. Sector susceptibility is calculated as the average over the susceptibility of each of the corresponding sector in each of the countries. Error bars show standard deviations. Source data are provided as a Source Data file.

Supplementary Figure 3

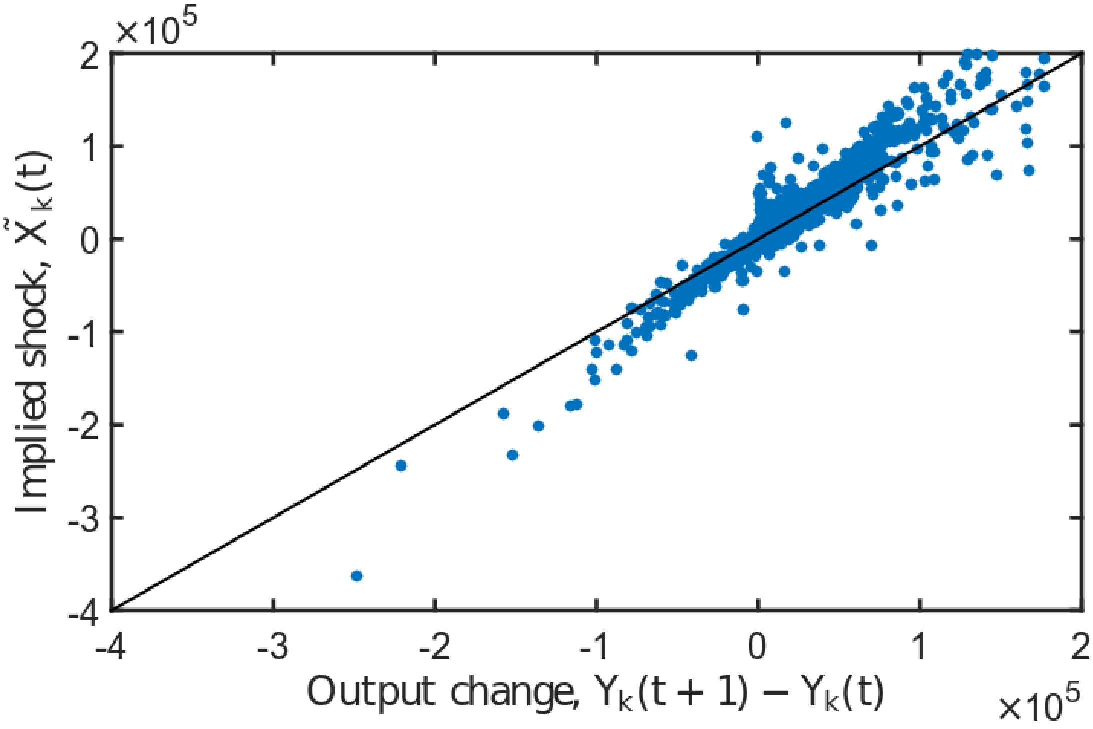

Observed and implied shocks. Comparison of the observed output changes, $Y_k(t+1) - Y_k(t)$, and the implied step demand shock $\tilde{X}_k(t)$. Each sector from each country is shown for each year between 2003 and 2014 as a blue circle, the black solid line shows $\tilde{X}_k(t) = Y_k(t+1) - Y_k(t)$. There is a clear tendency that the implied shocks are larger in absolute value than the observed output changes, meaning that the response formalism typically attenuates the initial shock. There are, however, outliers that defy this general tendency. For instance, $t = 2008$, the largest negative implied shock was observed for the manufacturing of coke sector in the US. Source data are provided as a Source Data file.

Supplementary Figure 4

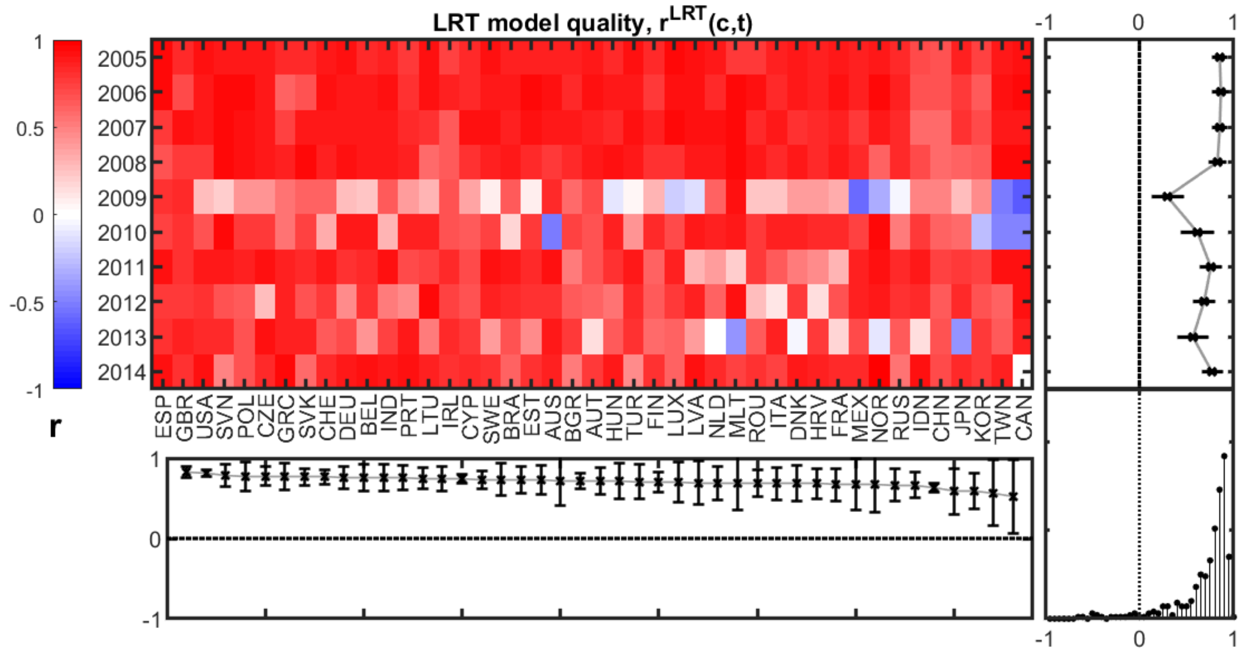

Quality of the LRT model. Values of the correlation coefficient $r^{\mathrm{LRT}}(c,t)$ between actual output changes and the predictions of the LRT model for each country c and year t (top left panel). We show $r^{\mathrm{LRT}}(c,t)$ averaged over each year (top right) and country (bottom left). Averages that are significantly different from zero are highlighted in bold and black. A histogram of $r^{\mathrm{LRT}}(c,t)$ over all countries and years (bottom right) shows the corresponding distribution. We find values of $r^{\mathrm{LRT}}(c,t)$ close to the maximal value of 1 for almost all countries and years except 2009. Source data are provided as a Source Data file.

Supplementary Figure 5

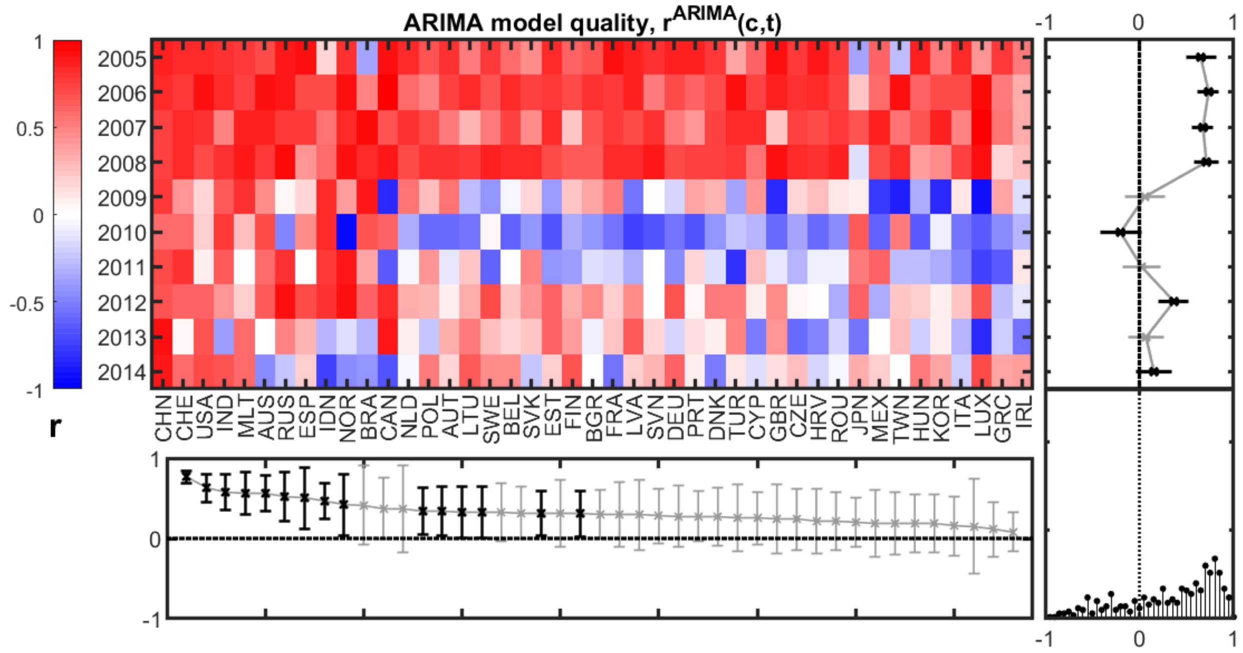

Quality of the ARIMA(1,1,1) model. Same as Supporting Fig. 5 for the correlation coefficient $r^{\mathrm{ARIMA}}(c,t)$ between actual output changes and the predictions of the ARIMA model. We find values of $r^{\mathrm{ARIMA}}(c,t)$ close to the maximal value of 1 up until 2008. In later years the average correlation coefficients fluctuate around zero. Source data are provided as a Source Data file.

Supplementary Figure 6

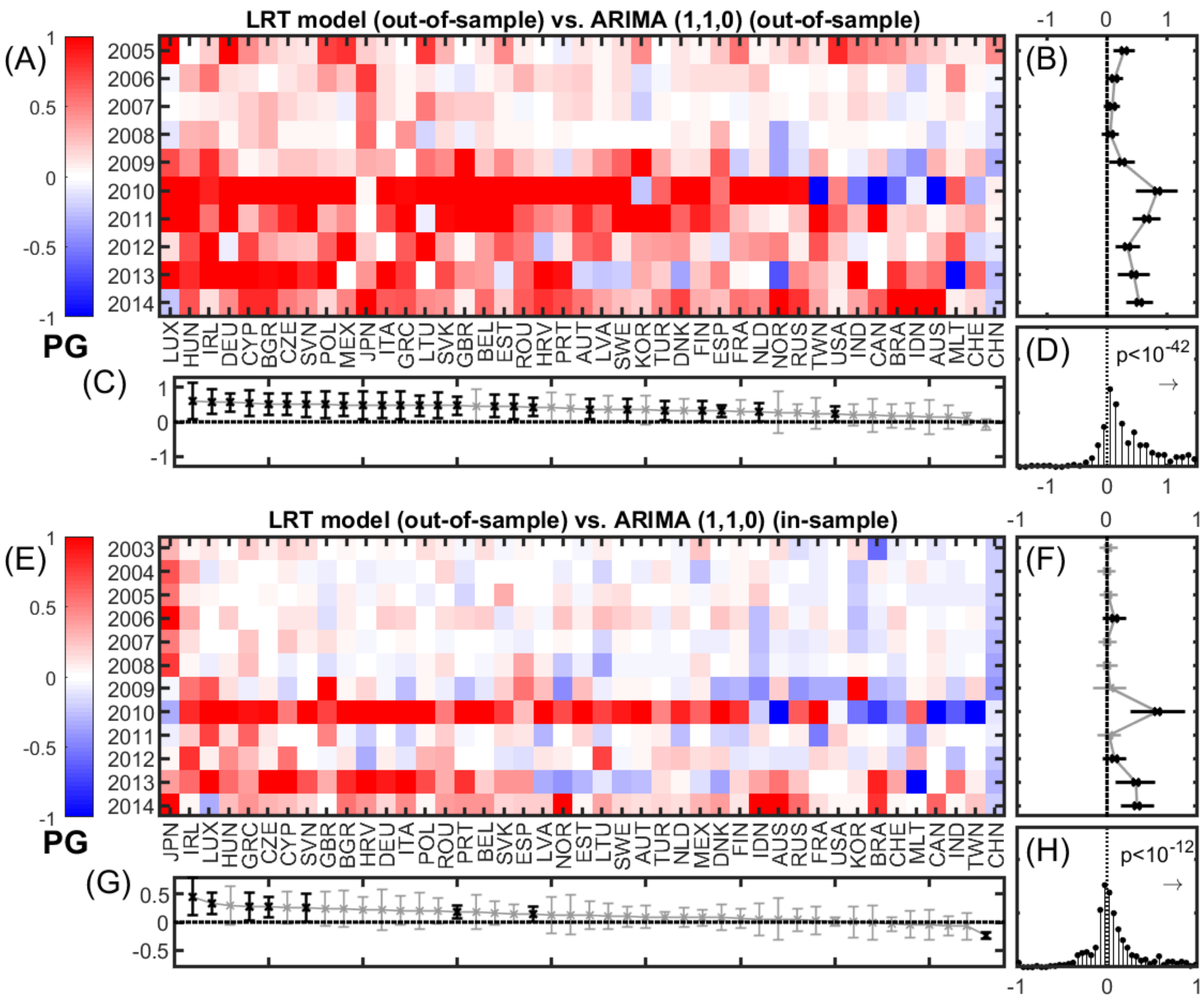

Comparison of the LRT and ARIMA(1,1,0) model. Same as Fig. 3 for comparison of the LRT versus an ARIMA(1,1,0) model. Source data are provided as a Source Data file.

Supplementary Figure 7

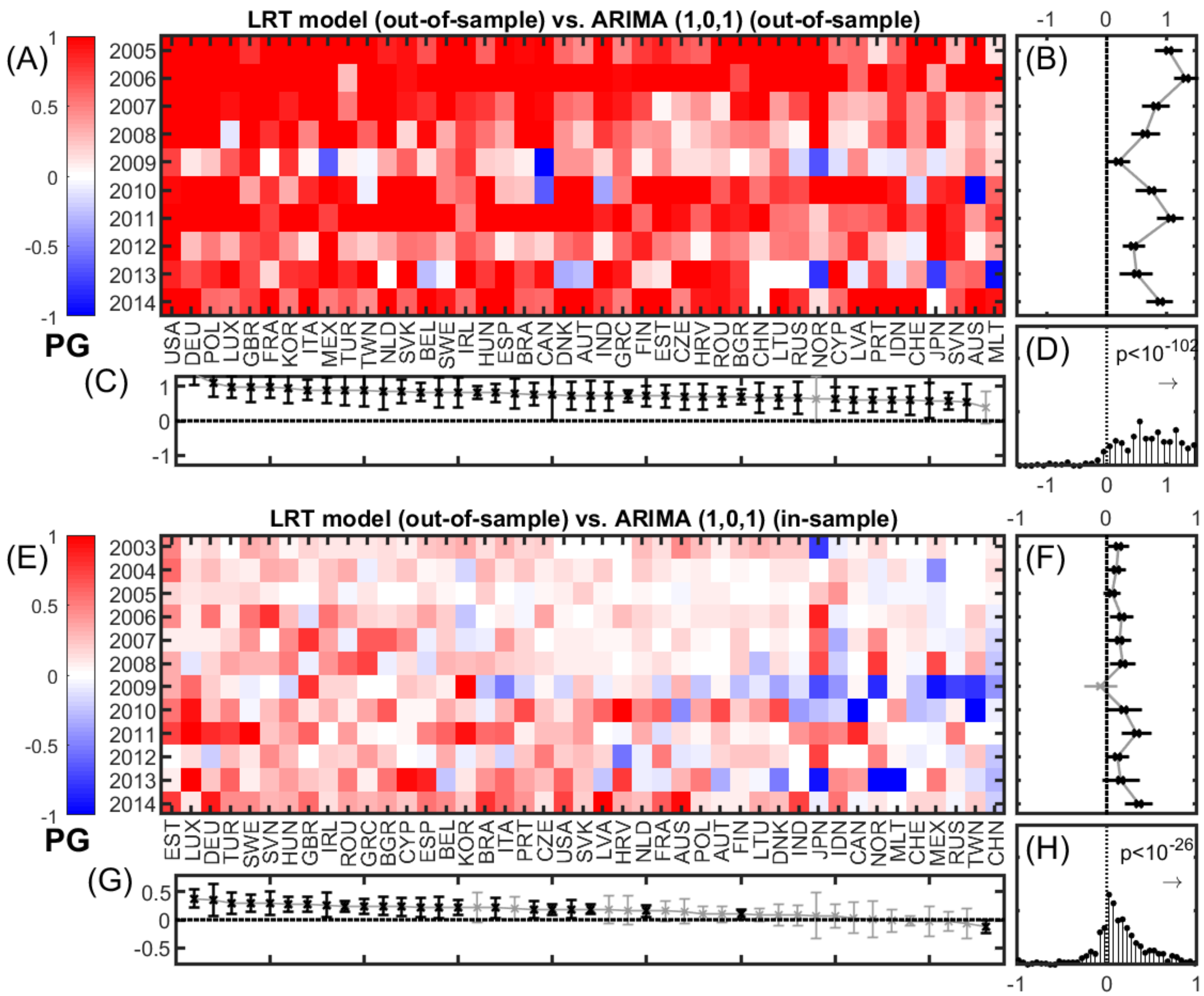

Comparison of the LRT and ARIMA(1,0,1) model. Same as Fig. 3 for comparison of the LRT versus an ARIMA(1,0,1) model. Source data are provided as a Source Data file.

Supplementary Figure 8

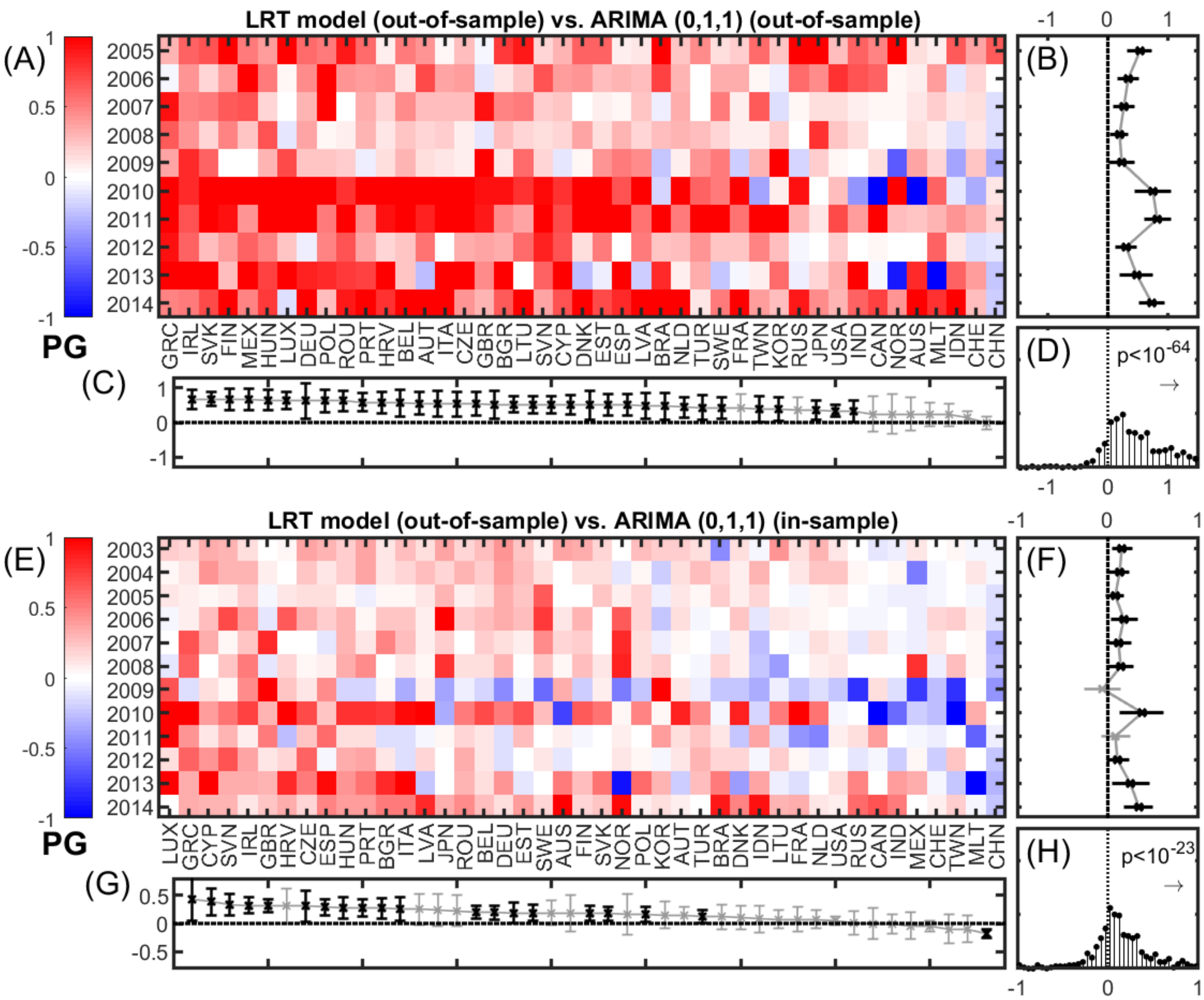

Comparison of the LRT and ARIMA(0,1,1) model. Same as Fig. 3 for comparison of the LRT versus an ARIMA(0,1,1) model. Source data are provided as a Source Data file.

Supplementary Figure 9

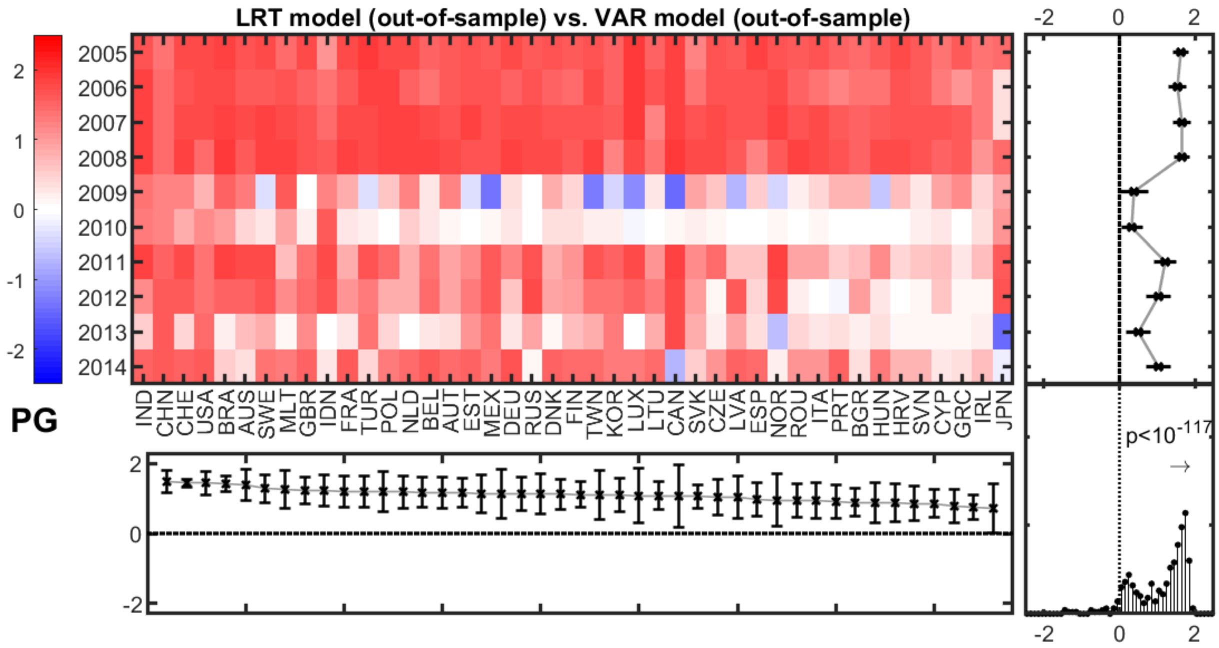

Comparison of the predictions of the linear response model with a sectoral VAR model. We compare the LRT model for a shock between years t and $t + 1$ with VAR models that have been calibrated using data from the year 2000. For each country and each year, we show the difference in Pearson's correlation coefficients between observed output changes and the different types of models, the predictability gain, PG. We show PG averaged over each year (panel to the right) and country (bottom panel). Here averages that are significantly different from zero are highlighted in bold and black. A histogram of PG over all countries and years (bottom right) shows the corresponding distribution. We find that in terms of predictability gain PG the LRT model is vastly superior to the VAR model ($p < 10^{-117}$). The advantage is least pronounced (but still significant) in 2009 and 2010, whereas values for PG in the other years are typically between one and two. Source data are provided as a Source Data file.

Supplementary Figure 10

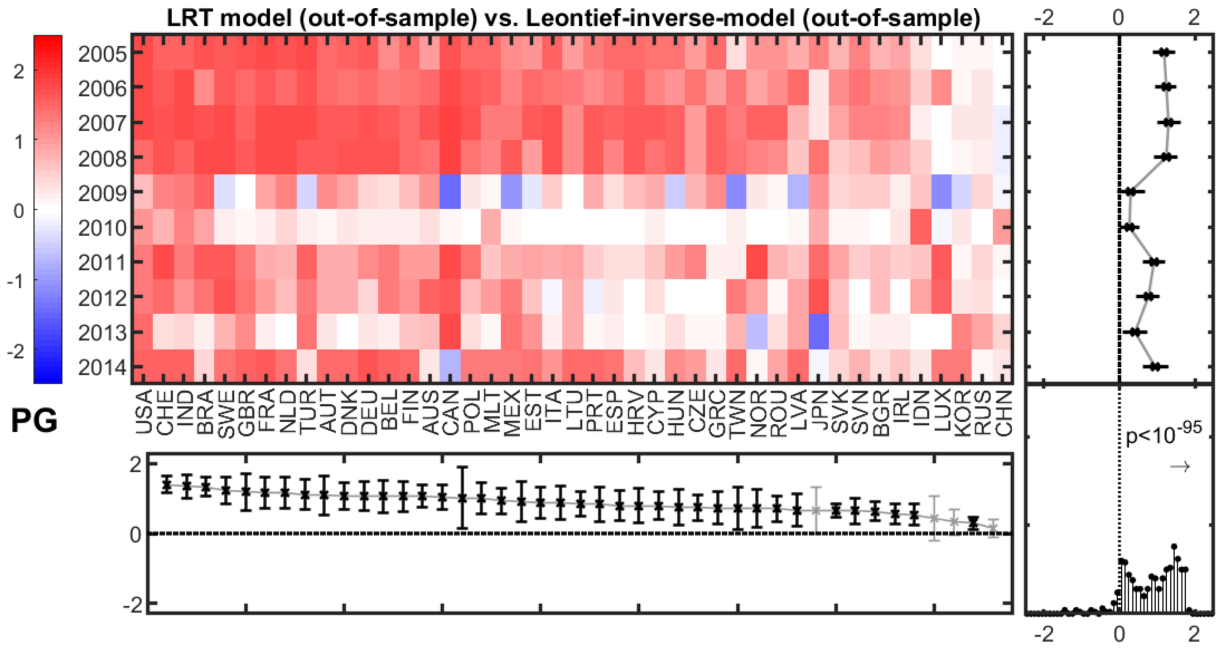

A comparison of the LRT model versus predictions from the perturbed equilibrium IO model. Same as in Supplementary Figure S9 with predictions from the perturbed equilibrium IO model (using implied shocks computed from LRT) replacing those of the VAR model. Source data are provided as a Source Data file.

Supplementary Table 1

Many phenomenological laws can be understood by linear response theory. Each of these laws is of the form $J = \rho X$ with X being a perturbation that induces a flux J. The size of the response J is proportional to the perturbation X. The linear dependence is described by transport coefficients ρ which can be a scalar, a vector, or a tensor.

**phenomenological law**	**perturbation X**	**induced flux J**	**transport coefficient ρ**
$I = \sigma V$	voltage V	current I	resistance σ
$S_{xy} = \sum_{kl} \mu_{ijkl} E_{kl}$	strain rate E	viscuous stress S	viscosity tensor μ
$B_i = \mu H_i$	magnetic field H	magnetic flux density B	magnetic susceptibility μ
$E_i = \epsilon_{ij} D_i$	electric field E	electric displacement D	electric susceptibility ϵ
$\Delta Y_k = \rho_{ki} X_i$	demand shock X	output change ΔY	economic susceptibility ρ

Supplementary Table 2

List of sectors included in the WIOD. For each sector we give its ISIC code(s), full name, short name, the type it was assigned to in Fig. 1, and its susceptibility score with 95% confidence interval.

code	full name	short name	type	susceptibility, ρ_i (95% CI)
A01	Crop and animal production, hunting and related service activities	Agriculture	Agriculture	0.0382 (0.0364–0.0404)
A02	Forestry and logging	Forestry	Agriculture	0.0290 (0.0270–0.0305)
A03	Fishing and aquaculture	Fishing	Agriculture	0.0183 (0.0177–0.0196)
B	Mining and quarrying	Mining	Mining	0.0568 (0.0505–0.0620)
C10-C12	Manufacture of food products, beverages and tobacco products	Food	Manufacturing	0.0355 (0.0336–0.0369)
C13-C15	Manufacture of textiles, wearing apparel and leather products	Textiles	Manufacturing	0.0226 (0.0213–0.0240)
C16	Manufacture of wood and of products of wood and cork, except furniture; manufacture of articles of straw and plaiting materials	Wood	Manufacturing	0.0270 (0.0259–0.0284)
C17	Manufacture of paper and paper products	Paper	Manufacturing	0.0295 (0.0267–0.0338)
C18	Printing and reproduction of recorded media	Printing	Manufacturing	0.0284 (0.0248–0.0319)
C19	Manufacture of coke and refined petroleum products	Coke	Manufacturing	0.0473 (0.0409–0.0519)
C20	Manufacture of chemicals and chemical products	Chemicals	Manufacturing	0.0450 (0.0428–0.0497)
C21	Manufacture of basic pharmaceutical products and preparations	Pharmaceuticals	Manufacturing	0.0191 (0.0184–0.0205)
C22	Manufacture of rubber and plastic products	Rubber	Manufacturing	0.0285 (0.0261–0.0314)
C23	Manufacture of other non-metallic mineral products	Mineral products	Manufacturing	0.0248 (0.0200–0.0309)
C24	Manufacture of basic metals	Metals	Manufacturing	0.0576 (0.0505–0.0643)
C25	Manufacture of fabricated metal products, exc. machinery & equipment	Metal products	Manufacturing	0.0387 (0.0361–0.0423)
C26	Manufacture of computer, electronic and optical products	Computer	Manufacturing	0.0292 (0.0275–0.0311)
C27	Manufacture of electrical equipment	Electricals	Manufacturing	0.0207 (0.0185–0.0241)
C28	Manufacture of machinery and equipment n.e.c.	Machinery	Manufacturing	0.0202 (0.181–0.0228)
C29	Manufacture of motor vehicles, trailers and semi-trailers	Motor vehicles	Manufacturing	0.0246 (0.0220–0.0267)
C30	Manufacture of other transport equipment	Transport equ.	Manufacturing	0.0127 (0.0112–0.0143)
C31-32	Manufacture of furniture; other manufacturing	Furniture	Manufacturing	0.0193 (0.0178–0.0208)
C33	Repair and installation of machinery and equipment	Repair	Manufacturing	0.0324 (0.0287–0.0348)
D35	Electricity, gas, steam and air conditioning supply	Electricity	Electricity & Water	0.0699 (0.0638–0.0736)
E36	Water collection, treatment and supply	Water	Electricity & Water	0.0183 (0.0175–0.0195)
E37-E39	Sewerage; waste collection, treatment and disposal activities; materials recovery; remediation activities and other waste management services	Waste	Electricity & Water	0.0275 (0.0256–0.0305)
F	Construction	Construction	Construction	0.0246 (0.0138–0.0305)
G45	Wholesale and retail trade and	Car trade	Trade	0.0322 (0-0307–0.347)

	repair of motor vehicles and motorcycles			
G46	Wholesale trade, except of motor vehicles and motorcycles	Wholesale trade	Trade	0.0866 (0.0808–0.0916)
G47	Retail trade, except of motor vehicles and motorcycles	Retail trade	Trade	0.0509 (0.0442–0.0553)
H49	Land transport and transport via pipelines	Land transport	Transport	0.0581 (0.0546–0.0620)
H50	Water transport	Water transport	Transport	0.0178 (0.0161–0.0199)
H51	Air transport	Air transport	Transport	0.0162 (0.0141-0.0180)
H52	Warehousing and support activities for transportation	Warehousing	Transport	0.0429 (0.0415–0.0445)
H53	Postal and courier activities	Post	Transport	0.0279 (0.0260–0.0296)
I	Accommodation and food service activities	Accommodation	Accommodation	0.0270 (0.0256–0.0284)
J58	Publishing activities	Publishing	Inform. & Comm.	0.0265 (0.0253–0.0277)
J59-J60	Motion picture, video and television programme production, sound recording and music publishing activities;	Entertainment	Inform. & Comm.	0.0335 (0.0316–0.0352)
J61	Telecommunications	Telecommunication	Inform. & Comm.	0.0398 (0.0338–0.0434)
J62-J63	Computer programming, consultancy and related activities	Computer programming	Inform. & Comm.	0.0298 (0.0277–0.0324)
K64	Financial service activities, exc. insurance and pension funding	Financial services	Finance	0.0656 (0.0630–0.0674)
K65	Insurance, reinsurance & pension funding, exc. compulsory social security	Insurance	Finance	0.0266 (0.0253–0.0275)
K66	Activities auxiliary to financial services and insurance activities	Auxiliary financial serv.	Finance	0.0379 (0.0366–0.0391)
L68	Real estate activities	Real estate	Other	0.0579 (0.0563–0.0592)
M69-M70	Legal and accounting activities; activities of head offices; management consultancy activities	Legal activities	Other	0.0634 (0.0589–0.0664)
M71	Architectural and engineering activities; technical testing and analysis	Architecture	Other	0.0390 (0.0373–0.0409)
M72	Scientific research and development	Research	Research	0.0148 (0.0024–0.0222)
M73	Advertising and market research	Advertising	Research	0.0375 (0.0344–0.0398)
M74-M75	Other professional, scientific and technical activities; veterinary activities	Other technical activities	Research	0.0311 (0.0299–0.0321)
N	Administrative and support service activities	Administration	Administration	0.0707 (0.0686–0.0727)
O84	Public administration and defence; compulsory social security	Public administration	Administration	0.0264 (0.0256–0.0272)
P85	Education	Education	Other	0.0235 (0.0226–0.0246)
Q	Human health and social work activities	Health	Other	0.0195 (0.0187–0.0206)
R-S	Other service activities	Other services	Other	0.0291 (0.0280–0.0301)
T	Activities of households as employers;	Household activities	Other	0.0272 (0.0259–0.0283)
U	Activities of extraterritorial organizations and bodies	Extraterrestrial org.	Other	-0.0396 (-0.2838–0.3025)

Supplementary Note 1

The basic idea of LRT is to compute the explicit time evolution of an external shock to a system in equilibrium. Originally, LRT has been formulated with thermal and mechanical transport processes in mind. The idea is that a thermodynamic system (e.g. a canister of gas, a block of metal, a piece of lumber, ...) can be prevented from relaxing to an equilibrium configuration by an external field. This field acts either directly on the system's microscopic constituents (think of an electric or magnetic field influencing the electrons in a block of metal) or that acts on the system boundaries (e.g. by generating a temperature gradient or by deforming the container of a system). In both situations the external field drives the system from equilibrium by means of induced transport processes. An electric field induces a current (e.g. transport of electrons) while a temperature gradient induces a heat flow (transport of particles with high kinetic energy). The central result of LRT is that a specific current (or flux) can be associated with such an external field. Furthermore, the magnitude of this flux is proportional to the magnitude of the external field.

Melville S. Green [22] and Ryogbo Kubo [23] independently found the exact mathematical expression that relates the field to its induced flux, the so-called transport coefficients ρ. These expressions are now called Green–Kubo relations. Note that ρ can be a scalar, a matrix, or a higher dimensional tensor, see Table S1. Their point of departure was that thermodynamic systems undergo a certain amount of fluctuations even at equilibrium. Given a perturbation in one of the microscopic degrees of freedom of the system (e.g. a fluctuation in a particle's velocity along a certain direction), the microscopic equations of motion allow to compute how strong and at which time other degrees of freedom (other particles) will "feel" this fluctuation. LRT assumes that other, larger perturbations will be felt in a similar way, i.e. with a similar delay and attenuation. Note that in many cases it is not even remotely possible to get a firm analytical understanding of the thermodynamics of a system once it is removed from equilibrium, given that it makes sense to talk about thermodynamics in such regimes at all. LRT provides one of the few analytic links that allows us to extrapolate out-of-equilibrium properties of a system from its behavior in equilibrium and therefore one of the paramount achievements of 20th century statistical mechanics.

Supplementary Note 2

In this work we use the World Input–Output Database (WIOD) release from November 2016 (http://www.wiod.org/release16). This release consists of several data tables that cover 28 EU countries and 15 other major countries (Australia, Brazil, Canada, China, India, Indonesia, Japan, Mexico, Norway, Russia, South Korea, Switzerland, Taiwan, Turkey, and the United States) from 2000 to 2014. The 15 non-European countries were chosen in order to cover all major parts of the world economy while ensuring that the data is available at a sufficient quality [36]. The resulting 43 countries account for more than 85% of world GDP [36]. For each year and country, WIOD contains data on 56 sectors according to the 2-digit ISIC revision 4 level.

The basic construction steps of the WIOD are as follows [36]. First, timeseries of national supply and use tables are constructed for each country based solely on national account statistics. With the use of international trade databases, these timeseries are disaggregated into imports by country of origin and use category. Finally, the resulting data items are integrated into a single input–output table. The construction of WIOD uses solely national account statistics as input for the construction of national supply and use tables. This ensures that (i) WIOD is fully consistent with national accounts and (ii) the use of a consistent methodology in categorizing products and services across time. This comes at the cost of a smaller coverage of countries and years with respect to other comparable databases that do not ensure such consistencies, see also [34] and [36].

Supplementary Note 3

ARIMA models are a quite general class of models for stationary timeseries, that is for timeseries that are characterized by statistical properties that do not change over time [37] (we only consider nonseasonable models here). Stationary timeseries show variations around their means that are of constant amplitude and that look similar over time. That is, given a snippet from a timeseries it is impossible to deduce when exactly the snippet was observed, e.g. after a couple of time steps or after observing the process for a million years. ARIMA models serve the purpose of forecasting timeseries based on observations of previous values that were collected within a specific time interval. It turns out that stationary timeseries have a quite limited set of properties that fully characterize them. These properties are encoded in the acronym ARIMA, which stands for auto-regressive integrated moving average. Auto-regression (AR) means that the next value of a timeseries can be predicted as a multiple of its prior values (maybe plus a constant term). Integration (I) means the timeseries should be forecasted by considering (also) *differences* between prior values, rather than (only) the values themselves. Moving average (MA) finally indicates that the timeseries is best predicted by considering not the last observed value (as in random walks without memory), but rather the average over several previously observed values. Each of these three properties, AR, I, and MA, can be present up to a specific order. For AR this order is the number p of auto-regressive terms, for I the number d of differences that are relevant, and for MA it is the number q of past observations that are included in the average. An ARIMA model is specified by the choice of these three numbers as an ARIMA(p,d,q) model. If the number of observations available to calibrate the ARIMA model is low (as it is the case for our work), it is often not meaningful to consider orders of parameters higher than one or two, as the corresponding coefficients for the higher order correction terms can not reliably be estimated.

Supplementary Note 4

Vector autoregression (VAR) models can be used to describe stochastic processes driven by linear dependencies on multiple other variables. In our case we can consider the sectoral outputs $Y_k^c(t)$ of country c as a stochastic process that may depend on the output of all other N sectors in the same country at the previous timestep, the outputs $Y_j^c(t-1)$ with $j \in \{1, \dots, N\}$. Note that ARIMA models assume that output changes in each sector can be predicted based on past values of the same sector only. By benchmarking the LRT model against a VAR model we can go beyond this limitation and test the LRT framework against a regression model that captures the structure of inter-industry dependencies in a more comprehensively way. The drawbacks of VAR models are that they do not scale well with system size. For a first order VAR model with $N = 56$ sectors one needs to estimate no less than $N^2 = 3{,}136$ different parameters. It is therefore completely hopeless to specify a sectoral VAR model from data given that we have only 15 observations for each sector. However, we can use a strategy to calibrate a sectoral VAR model similar to how we measured the response functions in the LRT framework. By assuming that the economy can be represented by the stochastic differential equation,

$$\dot{\mathbf{Y}} = (\mathbf{A} - \mathbb{I})\mathbf{Y} + \mathbf{D} + \mathbf{F}(t) \quad , \tag{11}$$

we generated 10,000 synthetic observations for the output of each sector. Using supercomputing resources (the Vienna Scientific Cluster 3, one of the hundred fastest computers worldwide (http://vsc.ac.at/systems/vsc-3/, accessed Sep 14, 2018.) we were then able to estimate the parameters of a first order VAR model using data from one year ($t = 2000$) for each country. By construction, the model has no linear trend. It remains to estimate the entries of the autoregression matrix $\mathbf{AR}^c$ for each country c, given by $\mathbf{Y}^c\,(t+1) = \mathbf{AR}^c\mathbf{Y}^c\,(t) + \mathbf{e}^c$ ($\mathbf{e}^c$ being the vector of intercepts). We then compare the forecasts of this VAR models with predictions of the LRT model, similar to the comparison with ARIMA models. The results are shown in Supplementary Figure S7. For almost all years and countries, the LRT model performs vastly superior than the VAR model ($p < 10^{-117}$). The advantage of the LRT model is least pronounced in the years 2009 and 2010 (where LRT still performs significantly better), whereas other years show predictability gains in the range between one and two. Note that these results are based on a VAR model calibrated in 2000 only due to the costly requirement for supercomputing resources. For a single country (Germany), we evaluated the VAR model also for several years afterwards and in particular for the crisis year 2008. Still, we found qualitatively the same result as shown in Supplementary Figure S7 with the majority of predictability gains lying in the range between one and two.

Supplementary Note 5

In this note we clarify the difference between the susceptibilty matrix ρ and the Leontief inverse matrix. In particular, for a step demand shock in LRT we find a non-equilibrium stationary state given by the linear relation $\langle\Delta\mathbf{Y}\rangle_{\mathrm{X}} = \boldsymbol{\rho}\mathbf{X}$, whereas in the standard Leontief IO economy we would expect a perturbed equilibrium state given by $\Delta\mathbf{Y} = (\mathbb{I} - \mathbf{A})^{-1}\mathbf{X}$. Wherein lies the fundamental difference between these two expressions?

To obtain the LRT solution for the output change, we describe the Leontief IO model in terms of a stochastic differential equation, see Eq. (5) in the main text,

$$\dot{\mathbf{Y}} = (\mathbf{A} - \mathbb{I})\mathbf{Y} + \mathbf{D} + \mathbf{X}(t) + \mathbf{F}(t) \quad . \tag{35}$$

The formal and stationary solution of this equation in the absence of a shock ($\mathbf{X}(t) = 0!$) is given in the Supporting Note S5, Eq.(32); it is a probability density function (where we changed variables to have a homogeneous equation, $\mathbf{Y} \to \mathbf{y} = \mathbf{Y} - (\mathbb{I} - \mathbf{A})^{-1}\mathbf{D}$),

$$\mathbf{f_0}(\mathbf{y}) = \frac{1}{\sqrt{(2\pi)^N|\boldsymbol{\sigma}|}} \exp\left(-\frac{1}{2}(\mathbf{y}\boldsymbol{\sigma}^{-1})\mathbf{y}\right) \quad . \tag{36}$$

The LRT prediction is an expectation value computed using this probability density, which is a multivariate normal distribution centred on the solution of the unperturbed Leontief IO model, $(\mathbb{I} - \mathbf{A})^{-1}\mathbf{D}$, when expressed in terms of the variable $\mathbf{Y}$. For a step demand shock we get Eq. (9) as stationary solution ($t \to \infty$),

$$\langle\Delta Y_k\rangle_{\mathrm{X}} = \rho_{ki}X_i, \text{ with } \rho_{ki} = \int_0^\infty (\sigma^{-1})_{ij}\langle Y_k(\tau)Y_j(0)\rangle_0 d\tau \quad , \tag{37}$$

where $\langle\cdot\rangle_0$ means that the expectation value is computed assuming $\mathbf{f_0}$ as underlying probability density function. We emphasize again that Eq. (37) shows how a non-equilibrium expectation value ($\langle\Delta Y_k\rangle_{\mathrm{X}}$ for $\mathbf{X} \neq \mathbf{0}$) can be defined in terms of a known equilibrium expectation value, namely ρ_{ki} in Eq. (37).

The computation of output changes in a perturbed Leontief IO model would proceed along a different route. Let us again consider a step demand shock $\mathbf{X}(t) = \mathbf{X}\theta(t)$. We are now interested in the equilibrium state of an economy under such a demand shock. Therefore, we introduce the perturbed demand, $\mathbf{D_P}$ as $\mathbf{D_P} = \mathbf{D} + \mathbf{X}$ and ask for the stationary solution of the stochastic differential equation,

$$\dot{\mathbf{Y}} = (\mathbf{A} - \mathbb{I})\mathbf{Y} + \mathbf{D_P} + \mathbf{F}(t) \quad . \tag{38}$$

Formally, Eqs. (35) and (38) are identical. However, the perturbed equilibrium perspective assumes a different stationary solution, namely

$$\mathbf{f_P}(\mathbf{z}) = \frac{1}{\sqrt{(2\pi)^N|\boldsymbol{\sigma}_\mathbf{P}|}} \exp\left(-\frac{1}{2}(\mathbf{z}\boldsymbol{\sigma}_\mathbf{P}{}^{-1})\mathbf{z}\right) \quad , \tag{39}$$

where $\mathbf{z} = \mathbf{Y} - (\mathbb{I} - \mathbf{A})^{-1}\mathbf{D_P} \neq \mathbf{Y} - (\mathbb{I} - \mathbf{A})^{-1}\mathbf{D}$ and $(\sigma_P)_{ij}(t) = \langle(z_i(t) - \langle z_i\rangle)(z_j(t) - \langle z_j\rangle)\rangle$

with $\boldsymbol{\sigma}_{\mathbf{P}}(t \to \infty) \equiv \boldsymbol{\sigma}_{\mathbf{P}}$. As could be expected, the stationary state of Eq.(38), i.e. the distribution of values of $\Delta\mathbf{Y}$ for $t \to \infty$, is now a multivariate normal distribution around the perturbed equilibrium state $(\mathbb{I} - \mathbf{A})^{-1}\mathbf{D}_{\mathbf{P}}$. It is clear that this solution coincides with the LRT solution for $\mathbf{D}_{\mathbf{P}} = \mathbf{D}$, i.e. in the absence of a shock, $\mathbf{X} = 0$. Also, only for $\mathbf{X} = 0$ would the correct expectation value to compute the susceptibility matrix and output change in LRT be given by the distribution $\mathbf{f}_{\mathbf{P}}(\mathbf{z})$.

Within this note we have now encountered three different types of expectation value, namely (i) the equilibrium expectation value using measure $\mathbf{f}_{\mathbf{0}}$, $\langle\cdot\rangle_0$, (ii) the perturbed equilibrium expectation value given by $\mathbf{f}_{\mathbf{P}}$, call it $\langle\cdot\rangle_{\mathrm{P}}$, and (iii) the nonequilibrium expectation value $\langle\cdot\rangle_{\mathrm{X}}$. These three expectation values coincide only in the absence of shocks, $\mathbf{X} = 0$. From a physical point of view, they describe three different types of system, namely a system relaxing to the state (i) $\mathbf{f}_{\mathbf{0}}$, (ii) $\mathbf{f}_{\mathbf{P}}$, or (iii) $\mathbf{f}_{\mathbf{0}}$ while responding to an external driving force, $\mathbf{X}(t)$ (a physicist would say that the external force $\mathbf{X}$ "does work on the system"). In physics, the latter class of systems are closely related to "dissipative structures", i.e. systems in a steady non-equilibrium state driven by an exchange of energy and/or matter with the environment.

In brief, while both the LRT and perturbed IO approach start from the same stochastic differential equation, Eqs. (35) and (38), they fundamentally differ in their definitions of expectation values. In constrast to expected values in the Leontief IO model in the perturbed equilibrium approach, the LRT approach assumes that the stationary solution of the system does *not* change after the application of an external shock. It might also be instructive to compare predictions of the LRT model with those from the perturbed Leontief IO model, i.e. the prediction $\langle\Delta Y(t + \Delta t)\rangle_{\mathrm{P}} = (\mathbb{I} - \mathbf{A})^{-1}\widetilde{\mathbf{X}}$, where $\widetilde{\mathbf{X}}$ is the implied shock from Eq. (2). We have to emphasize that such a comparison is problematic, as the use of the implied shock is only properly defined within the LRT framework, whereas the forecast using the perturbed IO model assumes a different type of dynamical system, as described above. In this sense, these two models are incompatible and should not be treated on equal footing as we do it here. Nevertheless, using the same evaluation strategy as we did for comparisons with time series models, we find that the LRT predictions significantly outperform predictions from the perturbed IO model ($p < 10^{-90}$); see also the Supplementary Figure 10.

Supplementary Note 6

Let $y \equiv \{y_i(t)\}, i = 1, \dots, N$ be the stochastic variables describing the time evolution of a dynamical system. In the following we assume the system to be linear and time invariant. Linearity means that the time evolution of the system is governed by a linear operator. Time invariance means that the response of the system does not depend on the time at which we apply the shock. We describe the state of the system by its probability density function, $f(y,t)$. The time evolution of the system is described by a linear time operator, $L_0(y)$, for instance a Fokker-Planck operator. In general, $L_0(y)$ can be any linear operator with a stationary solution for $f(y,t)$ for the LRT framework to be applicable. Further, the system is perturbed by a time-depending external field $X(t)$, i.e. we allow the external field to vary arbitrarily over time but not as a function of the stochastic variables y.. Under perturbation, the time evolution of $f(y,t)$ is then described by a perturbed operator $\Lambda_{\mathrm{X}}(y,t)$, as

$$\Lambda_{\mathrm{X}}(y,t) = L_0(y) + L_{\mathrm{X}}(y,t) \quad , \tag{12}$$
$$L_{\mathrm{X}}(y,t) = L_{\mathrm{X}}(y)X(t) \quad , \tag{13}$$

such that

$$\frac{\partial f}{\partial t} = L_0(y,t)f(y,t) \quad . \tag{14}$$

Let us denote the stationary solution for L_0 by $f_0(y)$, given by $L_0(y)f_0(y) = 0$.

Consider a small perturbation of the stationary solution of the form $f(y,t) = f_0(y) + \Delta f(y,t)$. For the time evolution we have then

$$\dot{f}(y,t) = \Delta\dot{f}(y,t) = \Lambda_{\mathrm{X}}(y,t)f(y,t) \tag{15}$$
$$= (L_0(y) + L_{\mathrm{X}}(y,t))(f_0(y) + \Delta f(y,t)) \tag{16}$$
$$= L_0(y)\Delta f(y,t) + L_{\mathrm{X}}(y,t)f_0(y) \quad . \tag{17}$$

The above equation can be solved using well-known properties of the Laplace transformation, given by $\tilde{f}(s) = \int_0^t dt\mathrm{e}^{-st}f(t)$. For the l.h.s. of the equation $\dot{f}(y,t) = L_0(y)\Delta f(y,t) + L_X(y,t)f_0(y)$ we get,

$$\dot{f}(y,t) = \Delta\dot{f}(y,t) = s\Delta\tilde{f}(s) - \Delta f(0) \quad , \tag{18}$$

where we can use that $\Delta f(0) = 0$. The r.h.s. simply transforms to $L_0(y)\Delta f(y,t) + L_{\mathrm{X}}(y,t)f_0(y) = L_0(y)\Delta\tilde{f}(s) + f_0(y)\tilde{L}_{\mathrm{X}}$. We obtain,

$$\Delta\tilde{f}(s) = \frac{\tilde{L}_{\mathrm{X}}f_0(y)}{s - L_0(y)} \quad . \tag{19}$$

Now we apply an inverse Laplace transformation to the l.h.s. and r.h.s. of Eq. 19. Therefore, we make use of the Laplace transform properties that for a product of functions we have $\tilde{f}(s)\tilde{g}(s) =$

$\int d\tau f(\tau)g(t-\tau)$ and that the exponential function, e^{at} transforms to $1/(s-a)$. Using these properties, Eq. 19 becomes

$$\Delta f(y,t) = \int_{-\infty}^{t} d\tau \ \mathrm{e}^{L_0(y)\cdot(t-\tau)} L_{\mathrm{X}}(\tau) f_0(y) \quad . \tag{20}$$

We are now interested in the expectation value of any dynamical variable $B(t)$ under the considered perturbations. Formally, we have

$$\langle B(t)\rangle = \int d^N y B(y) f(y,t) \tag{21}$$
$$= \langle B\rangle_0 + \int d^N y B(y) \Delta f(y,t) \quad . \tag{22}$$

By plugging in the solution for $\Delta f(y,t)$ obtained in Eq. 20 we get

$$\langle B(t)\rangle = \langle B\rangle_0 + \int_{-\infty}^{t} R_{B,X}(t-\tau) X(\tau) d\tau \quad , \tag{23}$$

where $R_{B,\mathrm{X}}(t)$ is the response function

$$R_{B,\mathrm{X}}(t) = \int d^N y \ B(y) \mathrm{e}^{L_0(y)t} L_{\mathrm{X}}(y) f_0(y) \ \ t \geq 0,$$
$$R_{B,\mathrm{X}}(t) = 0 \quad \mathrm{for} t < 0. \tag{24}$$

$R_{B,\mathrm{X}}(t)$ describes the response of the variable or observable $B(t)$ to the external force $X(t)$.

The response function can be expressed in terms of equilibrium correlation functions $c_{A,B}(\tau)$ between an observable $A(y(t))$ and $B(y(t))$ with lag τ. First, observe that

$$c_{A,B}(\tau) = \langle A(y(\tau)) B(y(0))\rangle \tag{25}$$
$$= \int d^N y \ d^N y_0 \ A(y) B(y_0) P(y,\tau;q_0,0) \quad ,$$

where $P(y,\tau;y_0,0)$ is the joint probability that the system is in configuration y_0 at time $t=0$ and in configuration y at t. This can be written as

$$P(y,\tau;y_0,0) = P(y,\tau|y_0,0) P(y_0,0) \quad . \tag{26}$$

Here, $P(y,\tau|y_0,0)$ is the conditional probability that the system evolved from state y_0 at $t=0$ to state y at t. Formally, this conditional probability is equal to the propagator $\mathrm{e}^{L_0(y)\cdot\tau}\delta(y-y_0)$. Note that $P(y_0,0)$ is the stationary solution $f_0(y_0)$. For the equilibrium correlation function this means that

$$c_{A,B}(\tau) = \int d^N y \ A(y) \mathrm{e}^{L_0(y)\cdot\tau} B(y) f_0(y) \quad . \tag{27}$$

Define the function $A(y)$ as $A(y) \equiv f_0^{-1}(y) L_{\mathrm{X}}(y) f_0(y)$ and introduce the generalized potential $\phi(y)$ through the relation

$$f_0(y) \equiv \mathcal{N} \mathrm{e}^{-\phi(y)} \quad , \tag{28}$$

with $\mathcal{N}$ as normalization constant. With these definitions we re-write the correlation function as

$$c_{B,A}(\tau) = \int d^N y \; B(y) \mathrm{e}^{L_0(y)t} L_{\mathrm{X}}(y) f_0(y) \quad , \tag{29}$$

and obtain the result that

$$c_{B,A}(\tau) = R_{B,\mathrm{X}}(\tau) \quad . \tag{30}$$

Consider a stochastic dynamical system with an external force $X_i(t)$ acting on component i and the stochastic force $F_{R,i}(t)$ with the properties $\langle F_{R,i}(t)\rangle = 0$ and $\langle F_{R,i}(t) \cdot F_{R,j}(t')\rangle = \epsilon_{ij}\delta(t - t')$. In particular, we consider linear systems of the type,

$$\dot{y}_i + \sum_{j=1}^{N} \gamma_{ij} y_j = F_{R,i}(t) + X_i(t) \quad . \tag{31}$$

Clearly, the stochastic IO dynamics that we consider in the main text is of the above type. To this end, note that one can always use a variable transformation of the form $Y \to y = Y - (\mathbb{I} - A)^{-1} D$ to rewrite the IO model into a homogeneous differential equation. From now on, we will use the summation convention. The covariance in the stationary regime (in the absence of external shocks $X(t)$) between $y_i(t)$ and $y_j(t)$ is denoted by $\sigma_{ij}(t) = \langle (y_i(t) - \langle y_i\rangle)(y_j(t) - \langle y_j\rangle)\rangle$, and $\sigma_{ij}(t \to \infty) \equiv \sigma_{ij}$. By the central limit theorem, the stationary solution for this process is immediately given by

$$f_0(y) = \frac{1}{\sqrt{(2\pi)^N |\sigma|}} \exp\left(-\frac{1}{2}(\sigma^{-1})_{ij} y_i y_j\right) \quad . \tag{32}$$

The perturbation is of the form $L_{\mathrm{X}}(y, t) = L_{\mathrm{X}}(y_i) X_i(t)$. We can identify the time evolution operator, $L_{\mathrm{X}}(y_i)$, and the generalized potential, ϕ, as

$$L_{\mathrm{X}}(y_i) = \frac{\partial}{\partial y_i} \; ; \quad \frac{\partial \phi}{\partial y_i} = (\sigma^{-1})_{ij} y_j \quad . \tag{33}$$

The response of component y_k to an external force acting on component i is then

$$R_{y_k,\mathrm{X}}(t) = (\sigma^{-1})_{ij} \langle y_k(t) y_j(0)\rangle \cdot X_i(t) \quad . \tag{34}$$

We derived these results making the following three assumptions, namely that our system is linear, time invariant and that the external field only depends on time. In addition, we only considered correction terms of first order in Eq. 17 and therefore neglect potential non-linear higher order effects. The applicability of the central limit theorem to the stationary solution is another consequence of these assumptions. Note that the fact that many economic time series follow fat-tailed or power law distributions is not at variance with the property that the stationay solution is given by a multivariate normal distribution. After all, there is no reason to assume that the stationary solution $f_0(y)$ can at any point in time be actually observed in the data due to the ceaseless impacts of direct and indirect shocks; $f_0(y)$ is a computational crutch.
